# A Novel Design Eco-friendly Microwave-assisted Cu–N@CQDs Sensor for the Quantification of Eravacycline via Spectrofluorimetric Method; Application to Greenness Assessments, Dosage Form and Biological Samples

**DOI:** 10.1007/s10895-023-03190-7

**Published:** 2023-03-03

**Authors:** Baher I. Salman

**Affiliations:** Pharmaceutical Analytical Chemistry Department, Faculty of Pharmacy, Al-Azhar University, Assiut Branch, Assiut, 71524 Egypt

**Keywords:** Eravacycline, Cu–N@CQDs, Human plasma, Milk samples, Fluorimetry

## Abstract

Community-acquired pneumonia is one of the most common infectious diseases and a substantial cause of mortality and morbidity worldwide. Therefore eravacycline (ERV) was approved by the FDA in 2018 for the treatment of acute bacterial skin infections, GIT infections, and community-acquired bacterial pneumonia caused by susceptible bacteria. Hence, a green highly sensitive, cost-effective, fast, and selective fluorimetric approach was developed for the estimation of ERV in milk, dosage form, content uniformity, and human plasma. The selective method is based on the utilization of plum juice and copper sulphate for the synthesis of green copper and nitrogen carbon dots (Cu–N@CDs) with high quantum yield. The quantum dots' fluorescence was enhanced after the addition of ERV. The calibration range was found to be in the range 1.0 – 80.0 ng mL^−1^ with LOQ equal to 0.14 ng mL^−1^ and LOD was found to be 0.05 ng mL^−1^. The creative method is simple to deploy in clinical labs and therapeutic drug health monitoring system. The current approach has been bioanalytically validated using US-FDA and validated ICH criteria. High-resolution transmission electron microscopy (HR-TEM), X-ray photon spectroscopy (XPS), Zeta potential measurements, fluorescence, UV–VIS, and FTIR spectroscopy have all been used to fully characterize the Cu–N@CQDs. The Cu–N@CQDs were effectively applied in human plasma and milk samples with a high percentage of recovery ranging from 97.00 to 98.80%.

**Supplementary Information:**

The online version contains supplementary material available at 10.1007/s10895-023-03190-7.

## Introduction

Community-acquired pneumonia is one of the most common infectious diseases and a substantial cause of mortality and morbidity worldwide. The need for innovative antibiotics is highlighted by the rise in the resistance of bacteria like Streptococcus pneumonia and Hemophilus influenza to beta-lactams, macrolides, and earlier generations of tetracyclines [1–3]. The widespread emergence of antibiotic resistance in recent years has resulted in the creation of new antibacterial medicines with distinct chemical structures. The FDA recently approved eravacycline (ERV) in 2018 for the treatment of acute bacterial infections and community-acquired pneumonia [1–3].

**Fig. 1:**
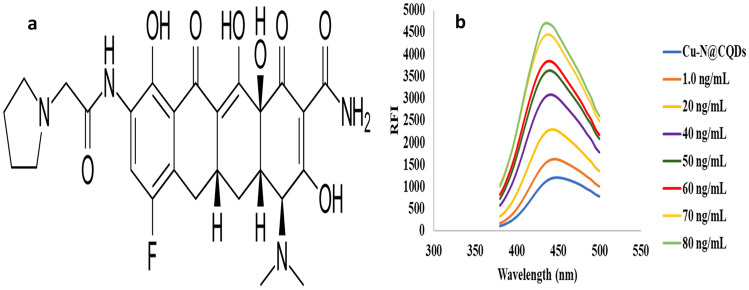
**a** Chemical structure of ERV, and **b** Reaction of ERV with Cu–N@CQDs at 445 nm (excitation at 360 nm)

Eravacycline (ERV) is a fluorinated tetracycline derivative. It has a broad spectrum against gram-positive and negative bacteria. Eravacycline is two to four times stronger than tigecycline against Gram-positive cocci and two to eight times stronger against Gram-negative bacilli [4]. **ERV** is [(4S,4aS,5aR,12aS)-4-(dimethylamino)-7- fluoro-3,10,12,12atetrahydroxy-1,11-dioxo-9-[2-(pyrrolidin-1-yl)acetamido]-1,4,4a,5,5a,6, 11,12a-octahydrotetracene-2-carboxamide] dihydrochloride as in Fig. 1a.

Only one chromatographic method has been reported for ERV estimation [5]. Various limitations were reported in the published method, such as using unavailable and expensive instruments, lacking different applications, and using organic solvents that affected the environment and increased environmental pollution [5].

Carbon quantum dots (CQDs) have become well-established as effective analytical sensors in the last 10 years due to their peculiar optical features, high water solubility, biocompatibility, non-toxicity, and ease of functionalization. CQDs are being positioned as an ideal alternative for fluorescent dyes and luminous derivatizing reagents due to their amazing and customizable fluorescence features [6–9].

In this work, eco-friendly, ultra-sensitive, selective green and the first fluorometric approach has been used to investigate ERV coupled with copper and nitrogen-doped carbon quantum dots (Cu–N@CQDs), which is sensitive and relatively selective via formation hydrogen bonding and electrostatic attraction based on the two highlighted, active, and strong neighboring hydrogen bonds between the hydroxyl/carboxyl of Cu–N@CQDs and fluorine atoms of ERV. The existence of carboxyl, hydroxyl, and copper groups led to viability to conjugate with the ERV drug [8, 10].

Transition metal nanostructures have prompted considerable interest due to their distinctive optical and electrical properties, as well as their excellent luminescent properties in semiconductor electroluminescence devices and fluorescence devices [8, 11].

In the last ten years, carbon quantum dots (CQDs), which have unique photocatalytic activity, excellent water solubility, biocompatibility, non-toxicity, and availability of functionalization, have solidified their position as a successful analytical sensor [6–9]. As a result, carbon quantum dots were used to analyze EVR in various applications, copper was introduced into the carbon dots synthesis to fine-tune the conduction band position of doped CQDs, which led to the adjustment of the functions used to treat the impurities and the fluorescence of doped carbon dots also increased its selectivity [10]. Implementing green analytical techniques has been one of analytical chemistry's main objectives over the last 20 years. The goal of green chemistry is to minimize or stop the use or production of dangerous pollutions. The development of analytical methods can be altered to conform to green chemistry principles in many ways, including sample size, sample preparation, and extraction methods. Green chemistry can be applied in a variety of ways, such as by redesigning experiments to use eco-friendly reagents or by managing waste. Numerous evaluation tools have been created to contrast the effectiveness of green chemistry methods with those of more conventional methods [9, 12].

Plum fruits are widely available around the world at cheap prices. The fruits are characterized by the presence of different active constituents such as vitamins A, B, and C, with high content of glucose, sucrose, phenolic compounds, and anthocyanin [13].

The purpose of this work is to develop an ultra-sensitive, quick, cost-effective, time-saving, and easy spectrofluorimetric approach for ERV estimation utilizing Cu–N@CQDs. The proposed method has been effectively applied to milk samples, pharmaceutical formulations, and human plasma. Furthermore, this study's greenness is consistent with global claims about green chemistry and safety.

## Experimental Part

### Materials and Reagents

Eravacycline (99.80%) powder and Xerava^®^ 50 mg vial were obtained from Tetraphase Pharmaceuticals, USA. Plums juice was purchased from the local market of Egypt. Copper sulphate and acetonitrile were obtained from the El-Nasr Company in Egypt. Human plasma samples were obtained from the Egyptian blood bank and stored at -24 until analysis.

### Instrumentations of the Cu–N@CQDs Method

The analytical study was carried out using the following equipment's: FS5 spectrofluorometer (Edinburgh, UK) with a 150 W xenon lamp source for excitation. Also, with a 1-cm quartz cell and connected to Fluoracle^®^ software. The slit widths were set to 2 nm and the scanning speed 1000 nm/min. Fourier transform infrared (FT-IR) for Cu–N@CQDs was reported a Nicolet™ iS™10 FTIR spectrometer in the wave number range 400–4000 cm^−1^. The powder X-ray diffractometer (PXRD) was scanned by a Philips X-ray diffractometer. High-resolution transmission electron microscope (HR-TEM) images were captured via a JEOL JEM-100CX II unit with tungsten EM filament 120 (USA). The dynamic light scattering measurements (DLS) were scanned by the Zetasizer Red badge instrument of ZEN 3600 (Malvern, UK). MFMI-100A (MED Future) microwave instrument (2450 MHZ 1000W) was designed for catalyzing organic synthesis and solvent extraction. JK-DMS-HP digital magnetic stirrer heating mixer, (China). Thermostatic ultrasonic sonication homogenizer (Shanghai- China). Metrohm 913 pH meter (USA). Sigma 2-16KHL laboratory centrifuge (Germany).

### Synthesis of (Cu–N@CQDs)

To synthesize copper and nitrogen carbon quantum dots (Cu–N@CQDs), 50 mL of plum juice were mixed with 200.0 mg of copper sulphate. The solution was transferred into 125-mL glass round-bottom flask with a lid and then placed into the microwave. Microwave source (700 W) 2450 MHz for 5 min until the brown solution was formed. The residue was dispersed and then sonicated for 20 min to remove large particles. The solution was filtered through a 0.45 μm cellulose membrane and centrifuged at 4000 rpm for 10 min. The supernatant was filtrated via 0.45 μm cellulose membrane. The yellow color solution was obtained and then lyophilized for Cu–N@CQDs characterizations. After that 17.0 mg of Cu–N@CQDs were dispersed into 100-mL ultrapure water for analytical procedure.

### Preparation of Standard Solution of ERV

To prepare the solution (50.0 µg mL^−1^), 5.0 mg of authentic powder of ERV were dissolved in 100 mL of ultrapure water. Further dilution to 100 mL with ultrapure water to prepare working solutions.

### Estimation of ERV Pharmaceutical Form

For the Xerava^®^ 50 mg vial, the vial was evacuated and accurately weighed. An amount of ERV powder equivalent to 10.0 mg was dissolved in 50 mL ultrapure water, mixed well, and then diluted to 100 mL with ultrapure water.

### Estimation of ERV in Plasma Samples

The plasma samples were prepared simply as follow**s:** different amounts of ERV solution (0.1 – 8.0 µg mL^−1^) were added to 300 µL of plasma into centrifugation tubes. Then, 1.0 mL of acetonitrile was applied as a protein-precipitating agent [8, 9]. After being vortexed for 30 s, the liquid was diluted to 10 mL with ultrapure water. After centrifuging the mixture for 20 min (4000 rpm), 1.0 mL of the supernatant was utilized in the analytical method.

### Quantification of ERV in Milk Samples

A half milliliter of the milk sample was mixed with 1.0 mL of acetonitrile as a protein precipitating agent. After that, a different concentration of ERV was added, and the mixture was stirring for 30 s before being diluted to 10 mL with ultrapure water. The mixture was centrifuged for 10 min at 3500 rpm. 1.0 mL of the supernatant was utilized for further examination [14]. The drug-free milk samples were prepared using the same steps without the addition of ERV.

### Analytical Procedure for Cu–N@CQDs

A half milliliter of Cu–N@CQDs (0.17 mg mL^−1^) was mixed with 1.5 mL of Britton-Robinson (BR) buffer (pH 7.5) into 10-mL volumetric flask, then 1.0 mL of working solution of ERV was added to obtain the final concentration range (1.0 – 80.0 ng mL^−1^). The flasks were completed by adding double distilled water to the mark. The fluorescence intensity was measured at λ_em_ 445 nm after 10 min (excitation 360 nm).

## Results and Discussion

ERV is classified as novel halide tetracycline derivative antibiotic with a unique chemical composition that manages GIT infections, skin infections, and community-acquired pneumonia [4]. Nanomaterials are intelligent materials with various uses in both business and biomedical and bioimaging research [15–18]. The fluorescent Cu–N@CQDs with high quantum yield were prepared by the microwave-assisted method from a green source as a result of decreasing the use of organic solvents and chemical compounds [9, 16].

Furthermore, the fluorometric approach is a highly selective, cost-effective, fast, and sensitive technique that is commonly used for medicinal compounds [9, 19]. The proposed method is effectively applied for the estimation of ERV in pharmaceutical dosage forms, human plasma, and milk samples in the calibration range 1.0–80.0 ng mL^−1^ at 445 nm (excitation at 360 nm) Fig. 1b.

The fluorescence of Cu–N@CQDs was improved by combining with ERV due to the hydrogen bonding and electron-donor–acceptor complex between Cu–N@CQDs and ERV, as well as the presence of carboxyl, hydroxyl, and copper moieties. Carboxyl or hydroxyl in Cu–N@CQD groups and ERV fluorine generate active and strong local hydrogen bonding [10, 20].

In addition, the electron-accepting copper representative stabilized by the carbon skeleton of Cu–N@CQDs and the electron-donating identity of ERV, which may enhance the conjugation of C = C bonds, as well as the combining effect of hydrogen bonding and the electron-donor–acceptor complex impact, increased the introduction of enormous fluorophores and chromophores. [8, 10, 20]

### Characterizations of Copper and Nitrogen-doped Carbon Quantum Dots (Cu–N@CQDs)

**Fig. 2:**
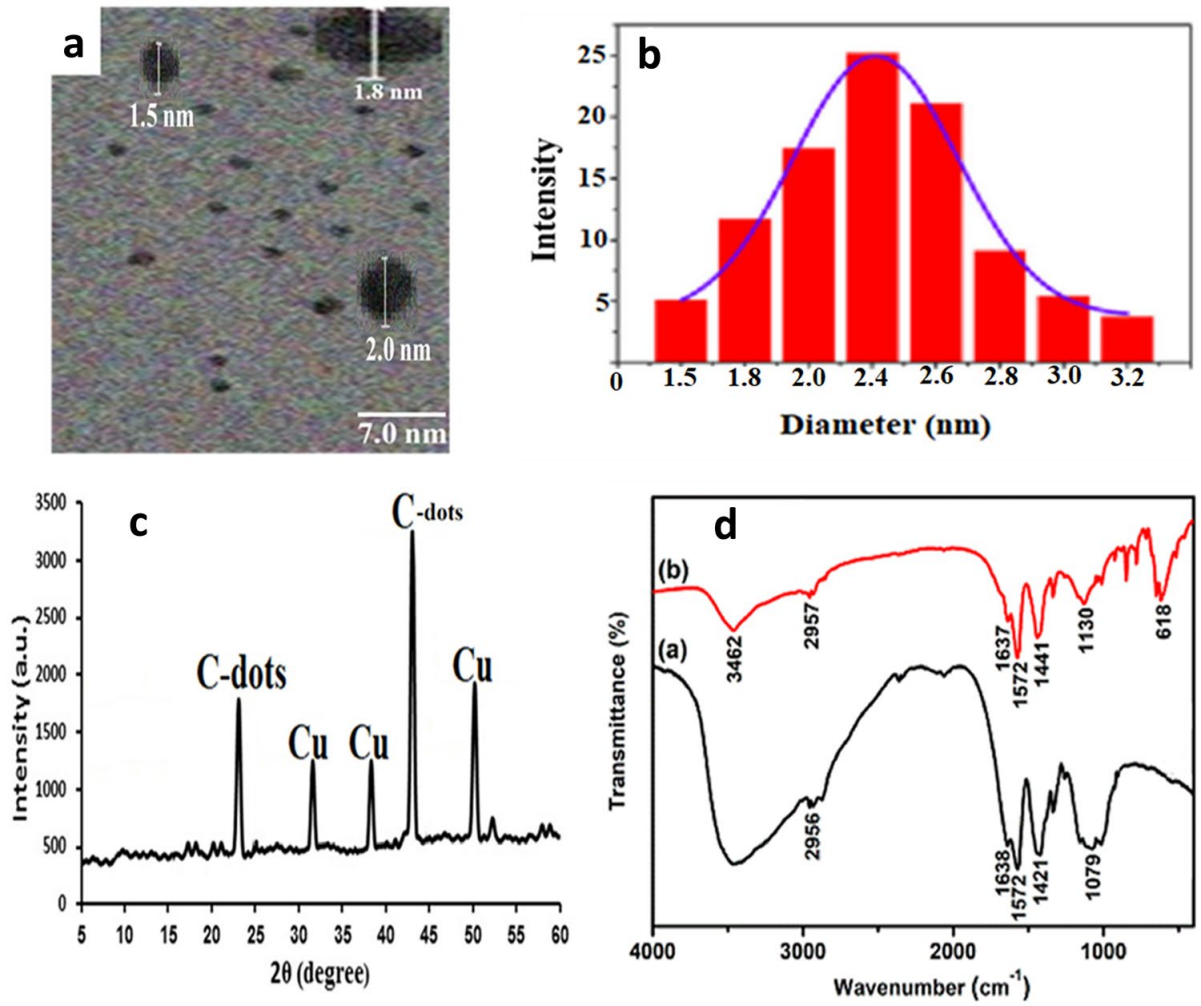
**a** High- Resolution transmission electron microscope image, **b** The dynamic light scattering for Cu–N@CQDs, **c** PXRD spectrum for Cu–N@CQDs, and **d** FTIR spectrum for N-CQDs (**a**), and for Cu–N@CQDs (**b**)

The morphology of Cu–N@CQDs was first studied by high-resolution transmission electron microscope (HR-TEM) where it exhibits a narrow size with an average diameter ranged from 1.5 to 2.0 nm as seen in Fig. 2a.

In addition, the particle size can be determined by measuring the random changes in the intensity of light scattered from the quantum dots solution using dynamic light scattering (DLS). Figure 2b shows the average size equal to 2.4 ± 0.16 nm which is slightly larger than TEM image because the larger molecules diffused slowly than smaller molecules [21]. Besides the variations in the size arise due to the random Brownian motion of the particles [21].

The PXRD pattern of Cu–N@CQDs is depicted in Fig. 2c. Two diffraction peaks are observed at 24.6° and 44.2° these peaks are associated with the amorphous carbon and graphitization carbon, respectively [19]. Different peaks of Cu doping in N@CQDs were observed as in Fig. 2c. Cu doping with N@CQDs was interpreted using the EDX spectrum for Cu–N@CQDs (Fig. S1). It was found identifiable peaks corresponding to C, N, O, and Cu were observed (Fig. S1).

The function groups on the surface of N@CQDs and Cu–N@CQDs were studied via the FTIR spectrum Fig. 2d.

The FTIR peaks appear at 3462, 2975, 1637, 1572, 1441 cm^−1^ corresponding to (NH, OH), CH_2_, C = O, (OH, NH stretching), C-O, and C-N respectively [19, 22]. Moreover, the peak intensities of the synthesized Cu–N@CQDs are weak compared with N-CQDs which refers to Cu–N@CQDs formation. In addition, the appearance of a peak at 618 cm^−1^ corresponds to Cu–O not found in the N@CQDs FTIR spectrum Fig. 2d.

Furthermore, X-Ray photoelectron spectroscopy (XPS) was performed for the elemental analysis. It was observed that the four peaks of Cu- N@CQDs at 285.80, 394.91, 539.40, and 932.11 eV correspond to C 1 s, N 1 s, O 1 s, and Cu respectively Fig. S2a.

The XPS of O 1 s as in Fig. S2b provides 2 characteristic peaks 530. 5 and 531.80 corresponding to Cu–O and C–O–C. In addition, four characteristic peaks for C 1 s were observed at 284.5, 285.2, 286.4, and 288.5 eV, due to the presence (C–C and C = C), C-N, C-O, and C = O groups, respectively Fig. S2c.

As shown in (Fig. S2d) two characteristic peaks were observed at 934.5 and 952.5 eV, which refer to Cu 2p_3/2_ of Cu^+2^ and Cu 2p_1/2_ of Cu^+^ [23].

### Calculation of the Quantum Yield Fluorescence of Cu–N@CQDs

The fluorescence quantum yield (QY) of Cu–N@QDs was studied via the single point method using the following literatures [8, 9]. Quinine sulphate was utilized as a reference, with a quantum yield of 54% in 0.1 M H_2_SO_4_ solution with a refractive index 1.33.

$${{\varvec{Q}}}_{{\varvec{N}}{\varvec{C}}{\varvec{Q}}{\varvec{D}}{\varvec{s}}}={{\varvec{Q}}}_{{\varvec{Q}}{\varvec{u}}{\varvec{i}}{\varvec{n}}{\varvec{i}}{\varvec{n}}}\times \frac{{{\varvec{F}}}_{{\varvec{N}}{\varvec{C}}{\varvec{Q}}{\varvec{D}}{\varvec{s}}}}{{{\varvec{F}}}_{{\varvec{Q}}{\varvec{u}}{\varvec{i}}{\varvec{n}}{\varvec{i}}{\varvec{n}}}} \times \frac{{{\varvec{A}}}_{{\varvec{s}}{\varvec{t}}}}{{{\varvec{A}}}_{{\varvec{N}}{\varvec{C}}{\varvec{Q}}{\varvec{D}}{\varvec{s}}}}\boldsymbol{ }\times \frac{{{\varvec{\eta}}}^{2}\boldsymbol{ }({\varvec{N}}{\varvec{C}}{\varvec{Q}}{\varvec{D}}{\varvec{s}})}{\boldsymbol{ }{{\varvec{\eta}}}^{2}\boldsymbol{ }({\varvec{Q}}{\varvec{u}}{\varvec{i}}{\varvec{n}}{\varvec{i}}{\varvec{n}})}$$

Q is the quantum yield while F is integrated fluorescence.

The proposed method achieves high quantum yield because shrinking the sizes of Cu–N@CQDs (1.8 nm) would increase quantum yields by generating more optical effects on the surface of quantum dots [24, 25]. The quantum yield of amine Cu–N doped quantum dots was found to be 40.20%.

### Optical Characters of the Green Synthesized Quantum Dots (Cu–N@CQDs)

**Fig. 3:**
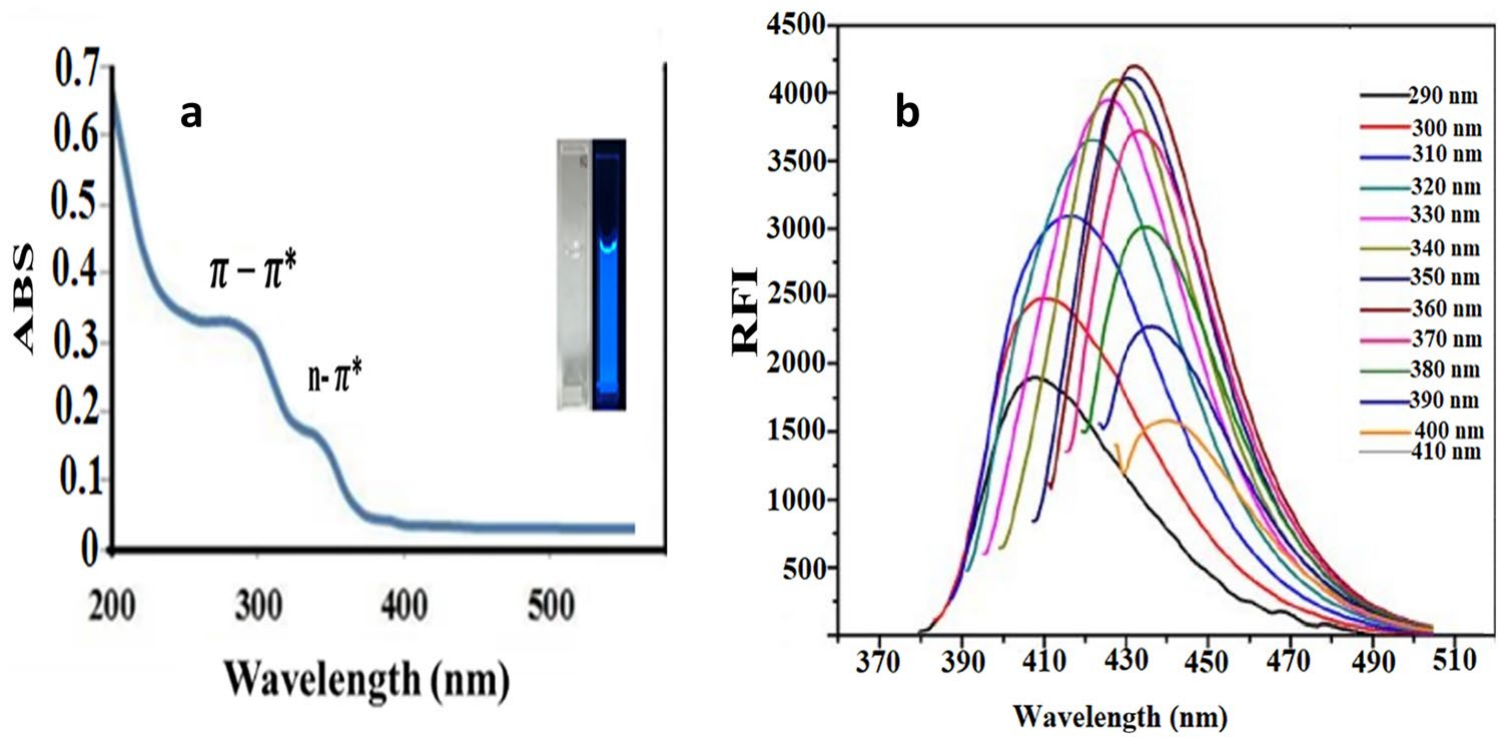
**a** UV spectrum for Cu–N@CQDs, and **b** Excitation dependent emission spectra for Cu–N@CQDs

The optical characters of the quantum dots were examined using UV spectra of Cu–N@CQDs. The quantum dots provide two absorption peaks at 290 and 330 nm as in Fig. 3a. The peaks correspond to π-π* electronic transition of C = C and n-π* electronic transition of C = O due to the amine on the surface of carbon quantum dots.

Different excitation wavelengths ranging from 290 to 410 nm were tested for excitation-dependent emission of Cu–N@CQDs. It was observed that increasing excitation wavelengths led to a red shift in the emission spectra followed by a decrease in RFI, confirming carbon dots' excitation-dependent emission Fig. 3b.

### The Optimization Process of Cu–N@CQDs

The sensitivity of the selected approach toward the reaction with ERV was enhanced via the optimization process (pH, volume of buffer, volume of Cu–N@CQDs, and reaction time).

**Fig. 4:**
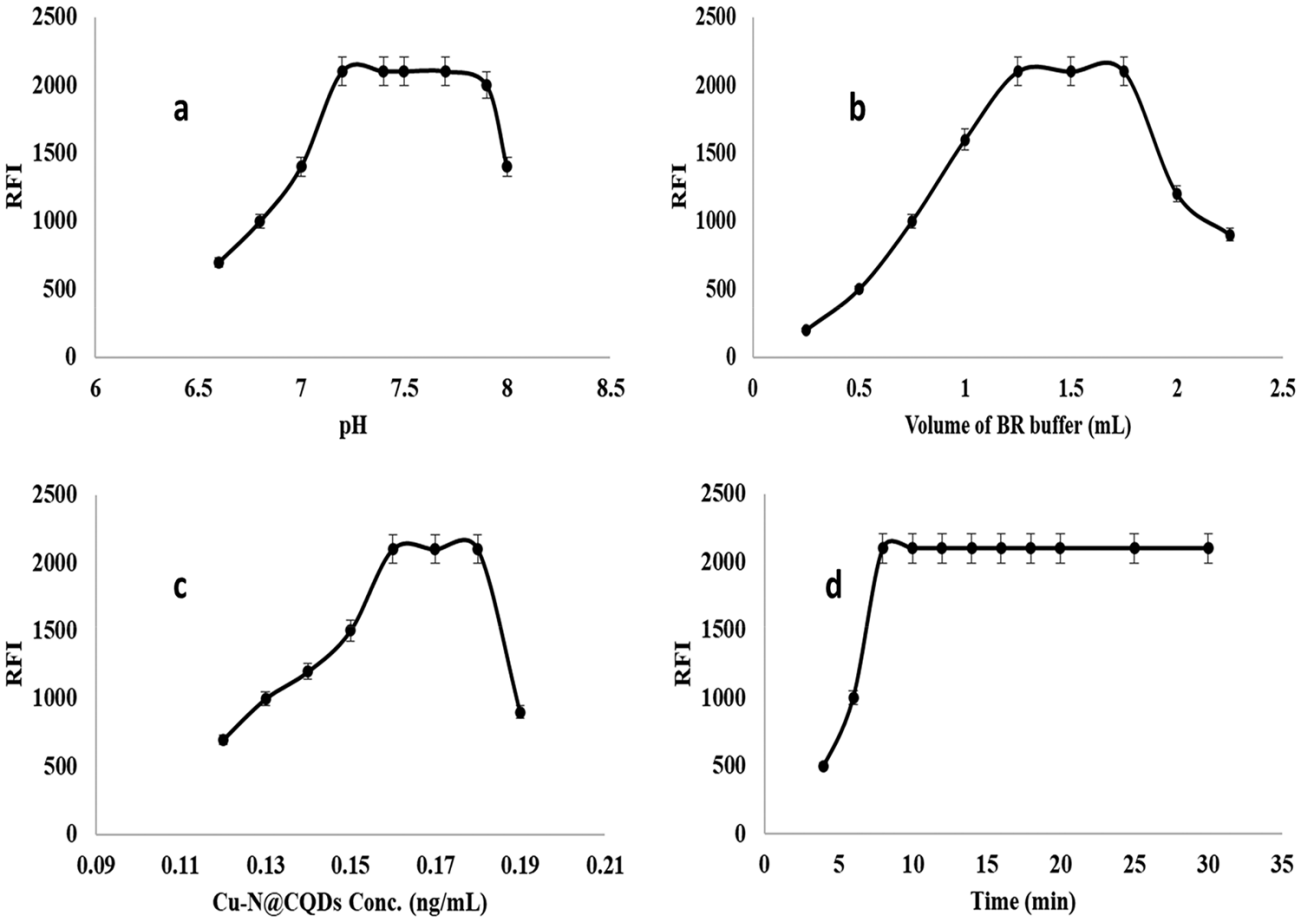
**a** Effect of pH range, **b** Effect of volume of buffer, **c** Effect of quantum dots concentration, and **d** Effect of reaction time using 20 ng mL^−1^ ERV

The effect of pH range on the fluorescence reaction of Cu–N@CQDs with ERV was investigated at different pH ranges from 6.5 to 8.1 The highest fluorescence response was obtained at pH 7.5 (Fig. 4a) using 1.5 ± 0.25 mL of BR buffer (Fig. 4b).

The effect of Cu–N@CQDs concentration on the fluorescence reaction with ERV was studied using different concentrations (0.11, 0.12, 0.13, 0.14, 0.15, 0.16, 0.17, 0.18, and 0.19 mg mL^−1^). The results observed in Fig. 4c refer to 0.17 mg mL^−1^ producing maximum fluorescence intensity.

The reaction time between ERV and Cu–N@CQDs was examined for 30 min. it was found the complete reaction was finished after 10 min. the increasing time than 10 min does not affect the RFI Fig. 4d.

### Validation of Cu–N@CQDs Method

The quantum dot method (Cu–N@CQDs) reaction with ERV was validated at optimum conditions using the International Conference of Harmonization (ICH) and bioanalytically validated via the US-Food and Drug Administration [26, 27]. The calibration curve was observed by plotting relative fluorescence intensity (RFI) vs ERV concentrations; the calibration range was set at 1.0 – 80.0 ng mL^−1^, as shown in Table 1. The detection (LOD) and quantitation levels (LOQ) were determined according to ICH guidelines [26] using the following formulations: LOQ = 10 ϭ/ slope, LOD = 3.3 ϭ/ slope (ϭ is the standard deviation). LOD and LOQ were found to be 0.05 and 0.14 ng mL^−1^, respectively, indicating the ultra-sensitivity of the proposed method as shown in Table 1.

**Table 1:** Quantification analytical parameters for analysis of ERV using Cu–N@CQDs

**Parameter**	**Results**
**λ**_**ex**_** (nm)**	360
**λ**_**em**_**(nm)**	445
**Concentration range (ng mL**^**−1**^**)**	- 80.0
**Determination coefficient (r**^**2**^**)**	0.9996
**Slope**	40.12
**Intercept**	1381.40
**SD the intercept (Sa)**	0.60
**LOD (ng mL**^**−1**^**)**	0.05
**LOQ (ng mL**^**−1**^**)**	0.14

***LOD*** lower limit of detection, ***LOQ*** lower limit of quantitation

The accuracy of the Cu–N@CQDs reaction with ERV was performed using five different concentrations within the calibration range (5.0, 10.0, 30.0, 50.0, and 70.0 ng mL^−1^). The recovery results fell between the range of 100.80% to 101.50%, with RSD values ranging from 0.22 to 1.00 indicating high accuracy of the Cu–N@CQDs method Table 2.

**Table 2:** Accuracy and precision of the quantum dots Cu–N@CQDs method with ERV

**Sample number**	**Taken Conc** **(ng mL**^**−1**^**)**	**Found Conc** **(ng mL**^**−1**^**)**	% Recovery ^*^ ± RSD
**1**	5.0	5.04	100.80 ± 0.69
**2**	10.0	10.13	101.30 ± 1.00
**3**	30.0	30.45	101.50 ± 0.70
**4**	50.0	50.50	101.00 ± 0.46
**5**	70.0	70.60	100.85 ± 0.22
**Intra-day** **precision**	20.0	20.14	100.70 ± 0.81
	40.0	40.33	100.82 ± 0.57
	60.0	61.40	102.33 ± 0.61
**Inter-day** **precision**	20.0	20.10	100.50 ± 0.77
	40.0	40.11	100.27 ± 0.79
	60.0	60.10	100.16 ± 0.45

***RSD*** Relative standard deviation

^*^ Average of three determinations

While the intra-day precision of reaction ERV with Cu–N@CQDs was examined using three concentration levels (20.0, 40.0, and 60.0 ng mL^−1^) at three replicate measurements. The inter-day precision was examined using three concentrations that were measured as three replicates over three days. The outcomes show very good repeatability and excellent precision of the quantum dots method Table 2.

The robustness of the suggested analytical approach was evaluated using minor adjustments in analytical procedure settings such as pH, the volume of buffer, reaction duration, and Cu–N@CQDs concentration. It was discovered that a slight modification in the analyzed parameters had no significant influence on technique performance Table 3.

**Table 3:** Robustness of the quantum dots (Cu–N@CQDs) reaction with (20 ng mL^−1^) ERV

**Variations**		
		**% Recovery**^**a**^** ± RSD**
**Optimum condition**		102.18 ± 0.40
**1- Effect of pH (BR buffer)**		
	7.3	100. 06 ± 0.19
	7.7	99.96 ± 0.37
**2- Volume of buffer (mL)**		
	1.25	100.10 ± 0.74
	1.75	100.21 ± 0.86
**3- Cu–N@CQDs concentration (mg mL**^**−1**^**)**		
	0.16	99.79 ± 0.61
	0.18	99.91 ± 0.56
**4- Reaction time (min)**		
	8	99.70 ± 0.78
	12	99.93 ± 0.66

^**a**^Mean of three determinations

The matrix effect on ERV was studied using Cu–N@CQDs by application of three different concentrations within the calibration range were utilized. The results refer to no interference from the matrix solution with ERV Table 4.

**Table 4:** Matrix effect of the Cu–N@CQDs for analysis ERV in human plasma

	**Intra-day assay(n = 6)**		**Inter-day assay(n = 18)**	
**Conc** **(ng mL**^**−1**^**)**	**Accuracy** **(%)**	**Precision** **(CV %)**	**Accuracy (%)**	**Precision (CV %)**
20	97.66	1.67	97.32	2.11
30	98.31	1.39	98.00	1.82
70	98.48	1.91	98.22	1.95

The stability of ERV in human plasma was studied as follows, three concentrations were used to investigate the stability of ERV in human plasma, including low-quality control (LQC 5.0 ng mL^−1^), medium-quality control (MQC 40.0 ng mL^−1^), and high-quality control (HQC 70.0 ng mL^−1^) levels. These concentrations were employed to measure stability for three freeze–thaw cycles (-24 °C), long-term stability (1 month at -24 °C), short-term stability (12 h at -24 °C), and post-preparative. The obtained recovery percentages ranged from 97.30 to 98.82%, which indicates to the high stability of ERV in human plasma. Table 5 provides all the findings from the analysis of the stability effect on human plasma.

**Table 5:** ERV stability in human plasma under different conditions using Cu–N@CQDs method

	**LQC** **5.0 ng mL**^**−1**^	**MQC** **40.0 ng mL**^**−1**^	**HQC** **70.0 ng mL**^**−1**^
Three Freeze–thaw cycle stability (-24 °C)	98.11 ± 0.87	97.99 ± 1.21	98.00 ± 1.00
Long-term stability (1 month at -24 °C)	97.44 ± 1.43	97.30 ± 1.32	97.91 ± 1.56
Short-term stability (12 h at -24 °C)	97.93 ± 1.51	97.87 ± 1.38	97.60 ± 1.70
Post-preparative stability (6 h at room temperature 25 °C)	97.62 ± 1.69	97.81 ± 1.69	98.05 ± 1.38
Post-preparative stability (12 h at room temperature 25 °C)	98.82 ± 0.89	98.52 ± 1.54	97.94 ± 2.23

Data presented as recovery (%) ± SD (n = 5)

### Selectivity of Cu–N@CQDs Reaction

**Fig. 5:**
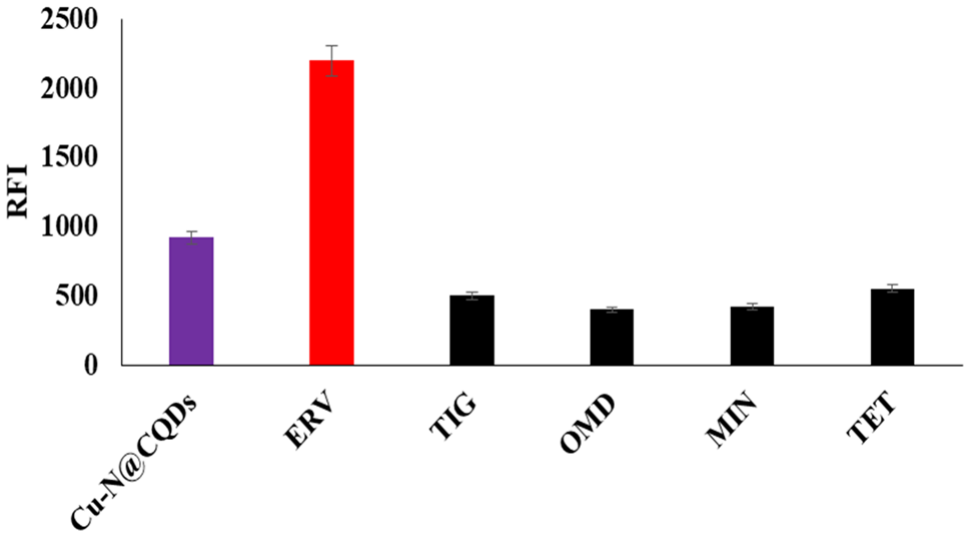
Selectivity study of the proposed method with different tetracycline derivatives (20 ng mL^−1^)

Various tetracycline derivative antibiotics with concentration (20 ng mL^−1^) such as tigecycline (TIG), omadacycline (OMD), minocycline (MIN), and tetracycline (TET) were utilized for selectivity study of the quantum dots method. As shown in Fig. 5, the Cu–N@CQDs fluorescence was enhanced with ERV due to the presence of fluorine which binds with Cu–N@CQDs forming a hydrogen bond with fluorescence enhancement. [8, 10, 20]

### Applications of Cu–N@CQDs in Human Plasma and Milk Samples

Different samples were examined using Cu–N@CQDs to boost the viability of the proposed sensors for ERV detection. The proposed method is effectively applied for the analysis of ERV in human plasma and milk samples with a high percentage of recovery compared with the reported method [5] Table 6.

**Table 6:** Application of the Cu–N@CQDs method for analysis of ERV in plasma and milk samples

	**Plasma**		**Milk**	
**Added conc** **(ng mL**^**−1**^**)**	**Found Conc** **(ng mL**^**−1**^**)**	% Recovery ^*^ ± RSD	**Found Conc** **(ng mL**^**−1**^**)**	% Recovery ^*^ ± RSD
1	0.98	98.00 ± 1.44	0.97	97.00 ± 1.78
5	4.86	97.20 ± 0.90	4.90	98.00 ± 0.94
10	9.78	97.80 ± 1.83	9.88	98.80 ± 0.62
50	48.77	97.54 ± 1.79	48.96	97.92 ± 0.80
70	88.18	98.11 ± 1.85	89.20	98.00 ± 1.59

^*^ Average of three determinations

### Applications in Pharmaceutical Dosage Form

The recommended method was used with effectiveness for quantifying ERV in commercial dosage forms (Xerava^®^ 50 mg vial). The percentage of recovery ± RSD was discovered to be 102.55 ± 0.65.

### Assessment of the Greenness of the Proposed Method

Recently, several assessment techniques for assessing the ecological effects of these analytical methodologies have been revealed. The evaluation of analytical techniques aids in reducing the environmental damage that these activities produce. For instance, a typical conventional HPLC machine generates 0.5L of organic waste each day [28], therefore, the greenness assessment became a must-do evaluation. Since the proposed method is suitable for application in the determination of ERV in various samples.

The Green Analytical Procedure Index (GAPI) was discoverd in 2018 [12], The GAPI offers 15 pictograms, each of which represents a stage within the primary 5 pentagrams and an analytical procedure. Red, yellow, and green colors serve as indicators for the color codes used in GAPI. The largest and lowest ecological impacts are denoted by the red and green colors, respectively. As shown in Table 7 except for milk and plasma samples, GAPI pictograms for the Cu-N@CQDs method only feature two red zones within the sampling pentagram, which represent off-line sampling and the requirement for the sample transportation. The distance between the sites for sample production or the clinical observation and the quality control (QC) laboratories result in off-line sampling, which in turn necessitates sample transportation. The GAPI pictograms of the Cu-N@CQDs method in milk and plasma samples have 3 red zones, 2 red zones within the sampling pentagram corresponding to the off-line sampling, and the need for sample transportation and the third red zone is corresponding to sample reparation as we use 1.0 mL of acetonitrile as an organic solvent in milk and plasma samples preparation.

**Table 7:** Greenness study of the proposed method for determination of ERV under different applications

	**Proposed method**	
**Technique**	Spectrofluorimetry	
**Application**	Milk and plasma samples	Pharmaceutical products
**Organic Solvents**	Acetonitrile	Totally Free
**Conditions**	Binding of ERV with copper and nitrogen doped carbon quantum dots (Cu–N@CQDs) in presence of Britton-Robinson buffer (pH 7.5)	
**Range**	1.0 – 80.0 ng mL^−1^	
**GAPI assessment**		
**AGREE assessment**		

On the other hand, AGREE [12, 29] is another assessment tool for evaluate the greenness that has been recently introduced on the color code based on GAPI. The main difference from GAPI is that it was based on the 12 basics of green analytical chemistry (GAC) [29]. AGREE shows a clock-shaped pictogram, in which the perimeter is divided into 12 sections, each corresponding to a GAC principle [29]. The center of the pictogram shows a numerical value estimating the ecological impact, where the closer to 1 refers to the better impact of greenness. As shown in Table 7, AGREE pictograms of the Cu-N@CQDs method in all applications except for milk and plasma samples show the lowest ecological impact, as expressed by the numerical evaluation. The perimeter of the proposed method is almost green, except for the third GAC principle due to off-line sampling which is unavoidable as pointed out in the GAPI pictogram discussion. The milk and plasma samples pictogram is also, almost green, except for the third GAC principle concerned with off-line sampling which is unavoidable. The slight ecological impact seen in the 11^th^, and 12^th^ principles of AGREE assessment arise from the use of ACN in plasma and milk samples extraction before analysis. The Cu-N@CQDs method for analysis of most samples would be green due to the absence of any required organic solvents. The use of low-energy spectrofluorometric equipment, its higher throughput, and simple sample preparation procedures without the need for derivatizing agents account for the better environmentally friendly behavior of the proposed methodology.

## Conclusion

The proposed study provides the first description of a spectrofluorimetric technique for the monitoring of the recently approved antibiotic medication eravacycline for community-acquired pneumonia. The reaction is based on green synthesis of high quantum yield Cu–N@CQDs. The method was effectively used for the estimation of ERV in human plasma, milk, and dosage form. In addition, the applications extend to greenness assessments with two greenness methods.

### Supplementary Information

Below is the link to the electronic supplementary material.

Supplementary file1 (DOCX 555 KB)

## Data Availability

All data generated or analyzed during this study are included in this published article (and its supplementary information files).
