# Supplementary material for: A Novel Design Eco-friendly Microwave-assisted Cu–N@CQDs Sensor for the Quantification of Eravacycline via Spectrofluorimetric Method; Application to Greenness Assessments, Dosage Form and Biological Samples
Source: J Fluoresc. 2023 Mar 3;33(5):1887–96. doi: 10.1007/s10895-023-03190-7 (PMC10539432; doi:10.1007/s10895-023-03190-7)
Supplement: Supplementary file 1 — Supplementary file1 (DOCX 555 KB) [file 10895_2023_3190_MOESM1_ESM.docx]

**A Novel Design Eco-Friendly Microwave-Assisted Cu-N@CQDs Sensor for the Quantification of Eravacycline via Spectrofluorimetric Method; Application to Greenness Assessments, Dosage Form and Biological samples**

Baher I. Salman

Pharmaceutical Analytical Chemistry Department, Faculty of Pharmacy, Al-Azhar University, Assiut branch, Assiut, 71524 Egypt, [bahersalman@azhar.edu.eg](mailto:bahersalman@azhar.edu.eg), [bahersalman2013@yahoo.com](mailto:bahersalman2013@yahoo.com)

**Tel.** +201099031345


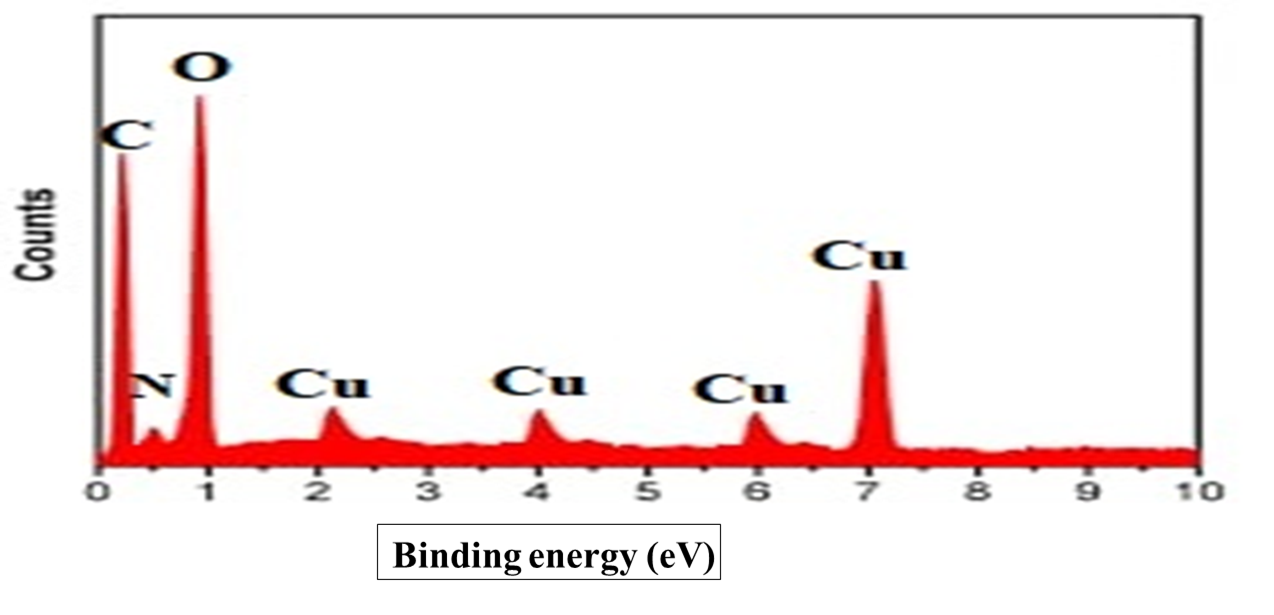


**Fig. S1** EDX spectrum of Cu-N@CQDs


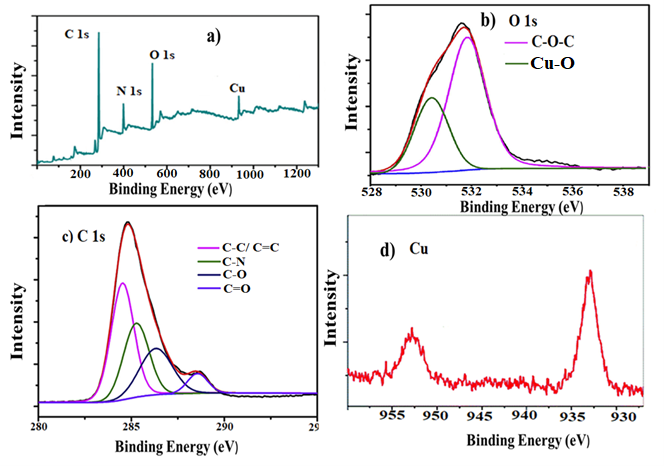


**Fig. S2** **a)** XPS spectrum for Cu-N@CQDs, **b)** O 1s spectrum, **c)** C 1s spectrum, and **d)** Cu spectrum of Cu-N@CQDs.
